# Supplementary material for: Assessment of RNA extraction protocols from cladocerans
Source: PLoS One. 2022 Apr 26;17(4):e0264989. doi: 10.1371/journal.pone.0264989 (PMC9041806; doi:10.1371/journal.pone.0264989)
Supplement: S2 File — (DOCX) [file pone.0264989.s002.docx]

**Assessment of RNA extraction protocols from Cladocerans**

**Muhammad Raznisyafiq Razak^a^, Ahmad Zaharin Aris^a,b*^, Fatimah Md Yusoff^b,c^, Zetty Norhana Balia Yusof^d,e^, Sang Don Kim^f^ and Kyoung-Woong Kim^f^**

*^a^* *Department of Environment, Faculty of Forestry and Environment, Universiti Putra Malaysia, 43400 UPM Serdang, Selangor, Malaysia*

*^b^ International Institute of Aquaculture and Aquatic Sciences, Universiti Putra Malaysia, 71050 Port Dickson, Negeri Sembilan, Malaysia*

*^c^ Department of Aquaculture, Faculty of Agriculture, Universiti Putra Malaysia, 43400 UPM Serdang, Selangor, Malaysia*

*^d^ Department of Biochemistry, Faculty of Biotechnology and Biomolecular Science, Universiti Putra Malaysia, 43400 UPM Serdang, Selangor, Malaysia*

*^e^ Institute of Bioscience, Universiti Putra Malaysia, 43400 UPM Serdang, Selangor, Malaysia*

*^f^ School of Earth Sciences and Environmental Engineering, Gwangju Institute of Science and Technology, 123 Cheomdangwagi-ro, Buk-gu, Gwangju, 61005, Republic of Korea*

***Corresponding author**

Ahmad Zaharin Aris

Tel: +603-89466732

Fax: +603-89438109

E-mail: zaharin@upm.edu.my


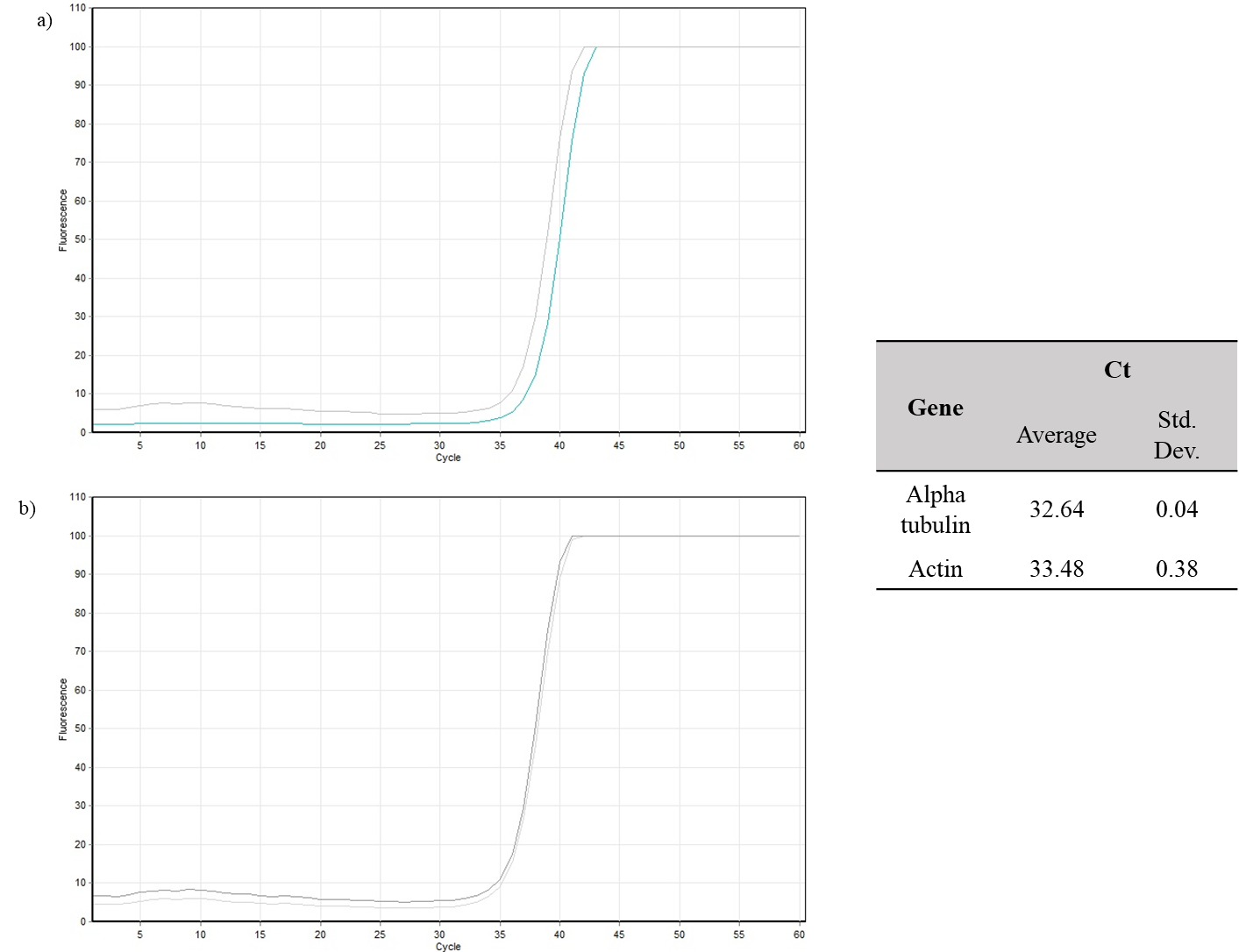


**Figure S1**: Real time PCR amplification cycle graphs of the amplicons of two *M. micrura* genes. Two (2) replicate of RNA extraction samples from the best extraction method (Method C) were used for cDNA synthesis. a) Alpha tubulin; b) Actin.


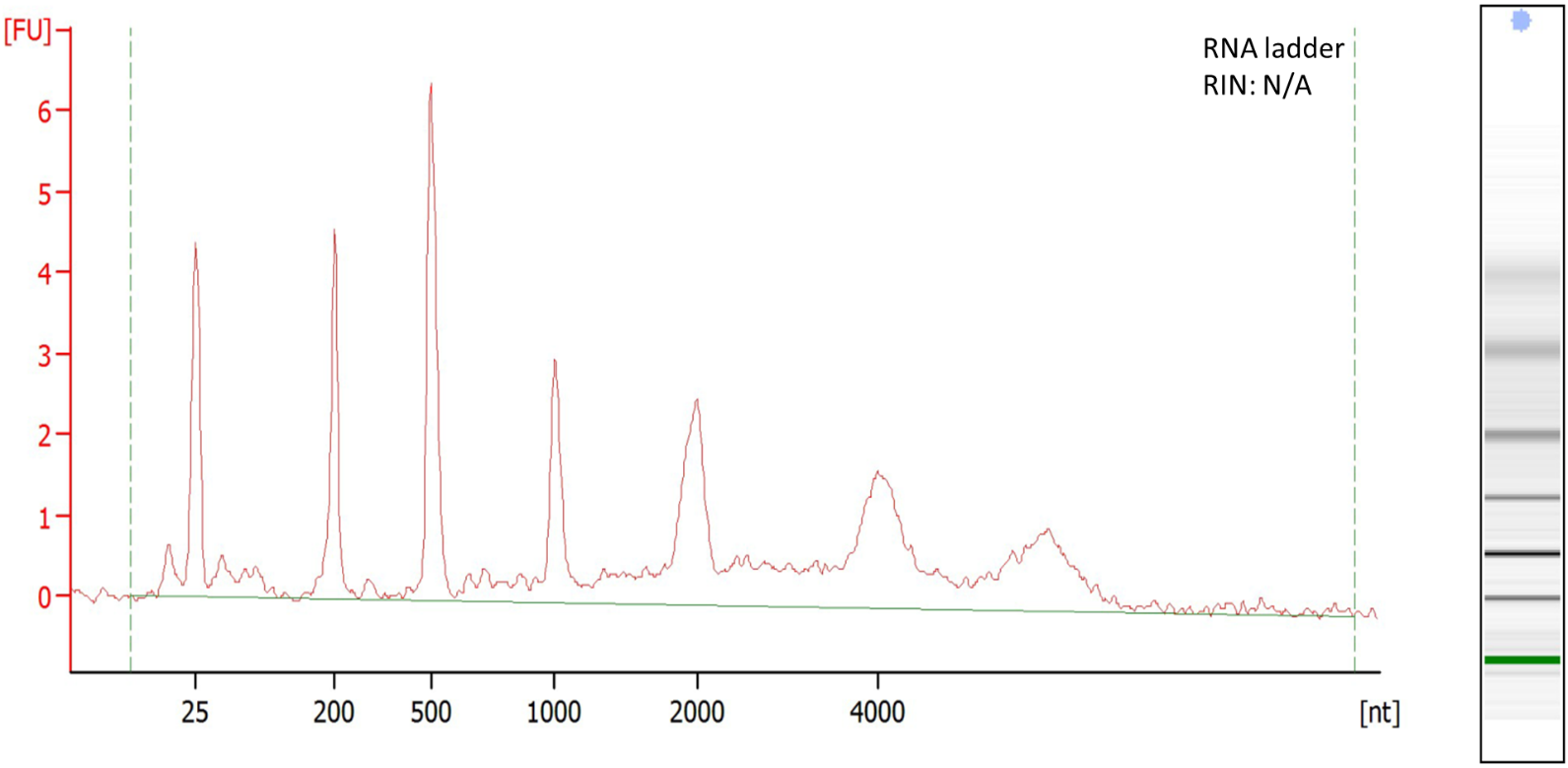


**Figure S2.** Electropherograms of RNA ladder. The integrity of RNA (RIN value) was measured by Agilent 2100 Bioanalyzer Agilent Technologies. The bioanalyzer instrument used Agilent RNA 6000 Pico Kit and supplied by Agilent Technologies. X –axis units in nt (Nucleotides); Y –axis units in FU (Fluorescence Units).


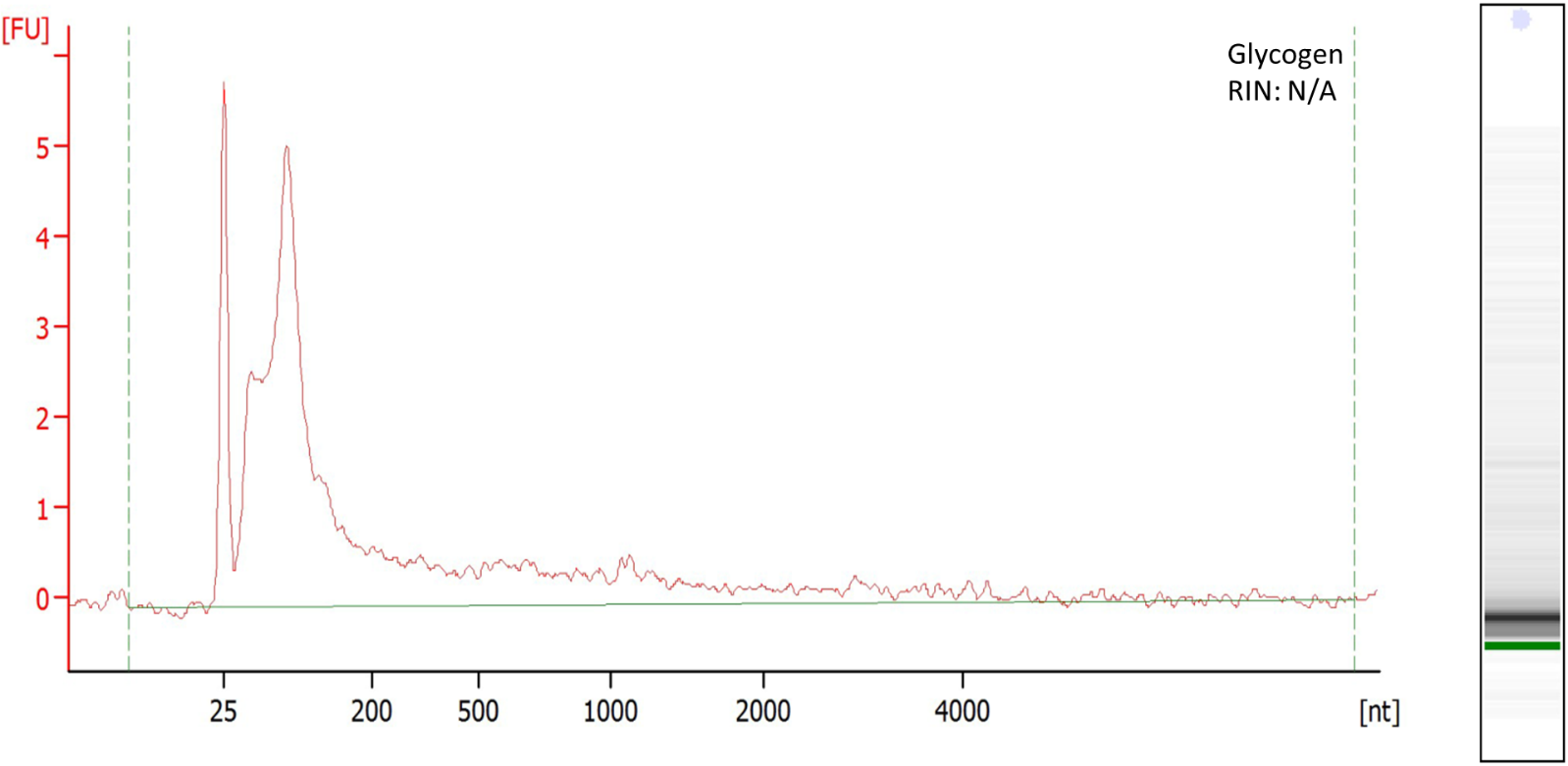


**Figure S3.** Electropherograms of glycogen. The integrity of RNA (RIN value) was measured by Agilent 2100 Bioanalyzer Agilent Technologies. The bioanalyzer instrument used Agilent RNA 6000 Pico Kit and supplied by Agilent Technologies. X –axis units in nt (Nucleotides); Y –axis units in FU (Fluorescence Units).


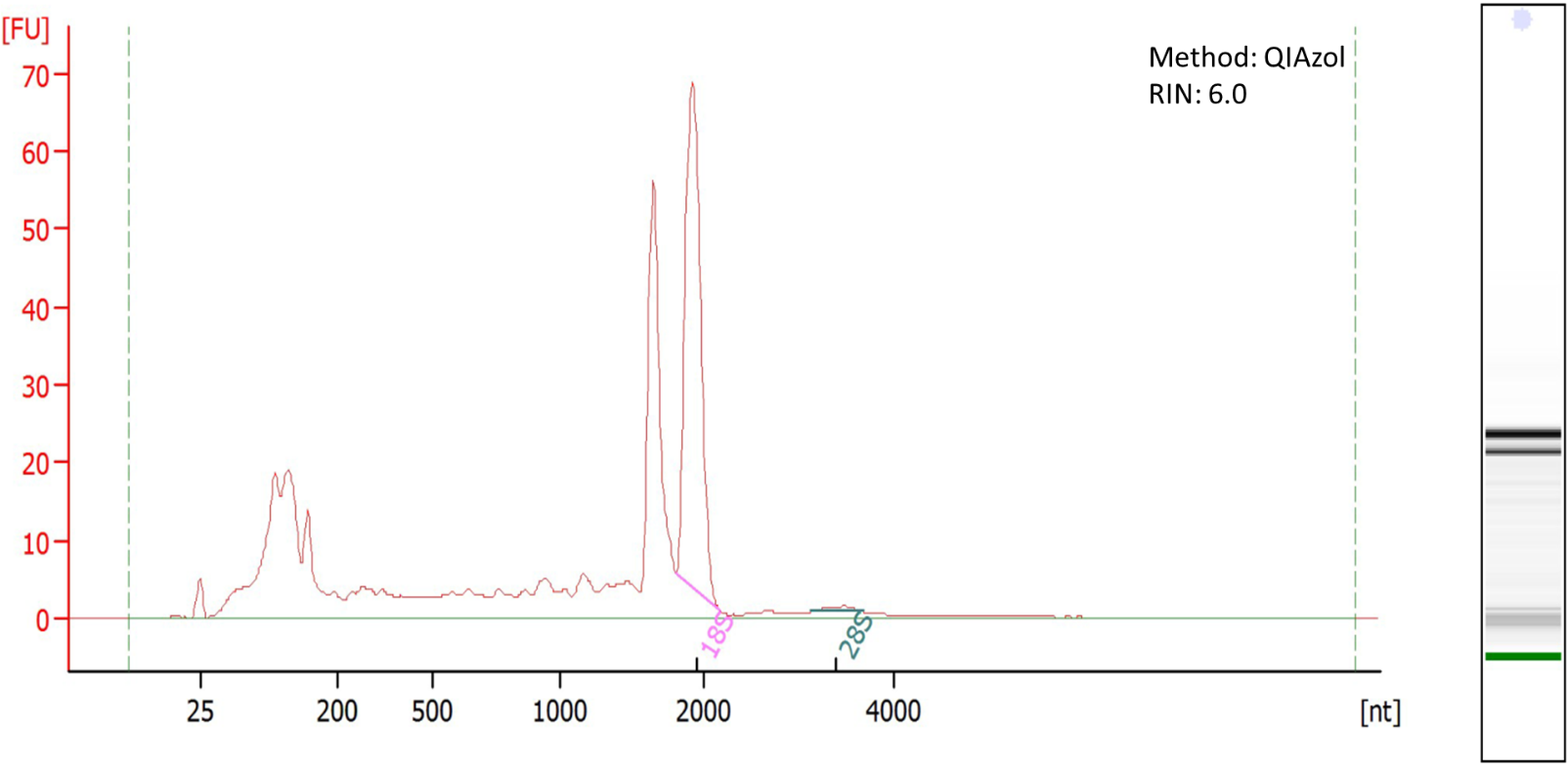


**Figure S4.** Electropherograms generated from *Moina micrura* RNA isolated using QIAzol method. The integrity of RNA (RIN value) was measured by Agilent 2100 Bioanalyzer Agilent Technologies. The bioanalyzer instrument used Agilent RNA 6000 Pico Kit and supplied by Agilent Technologies. X –axis units in nt (Nucleotides); Y –axis units in FU (Fluorescence Units).


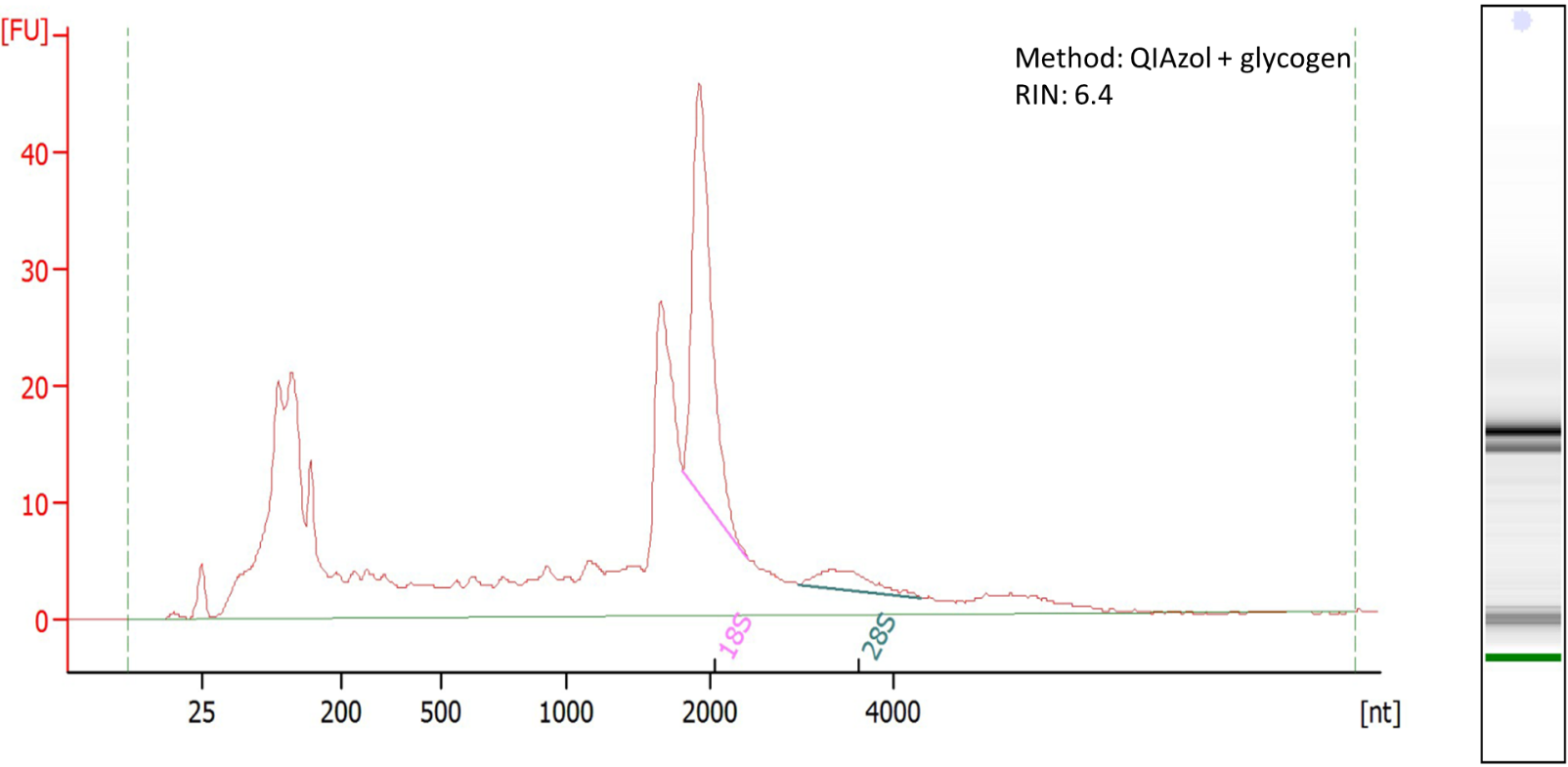


**Figure S5.** Electropherograms generated from *Moina micrura* RNA isolated using QIAzol + glycogen method. The integrity of RNA (RIN value) was measured by Agilent 2100 Bioanalyzer Agilent Technologies. The bioanalyzer instrument used Agilent RNA 6000 Pico Kit and supplied by Agilent Technologies. X –axis units in nt (Nucleotides); Y –axis units in FU (Fluorescence Units).


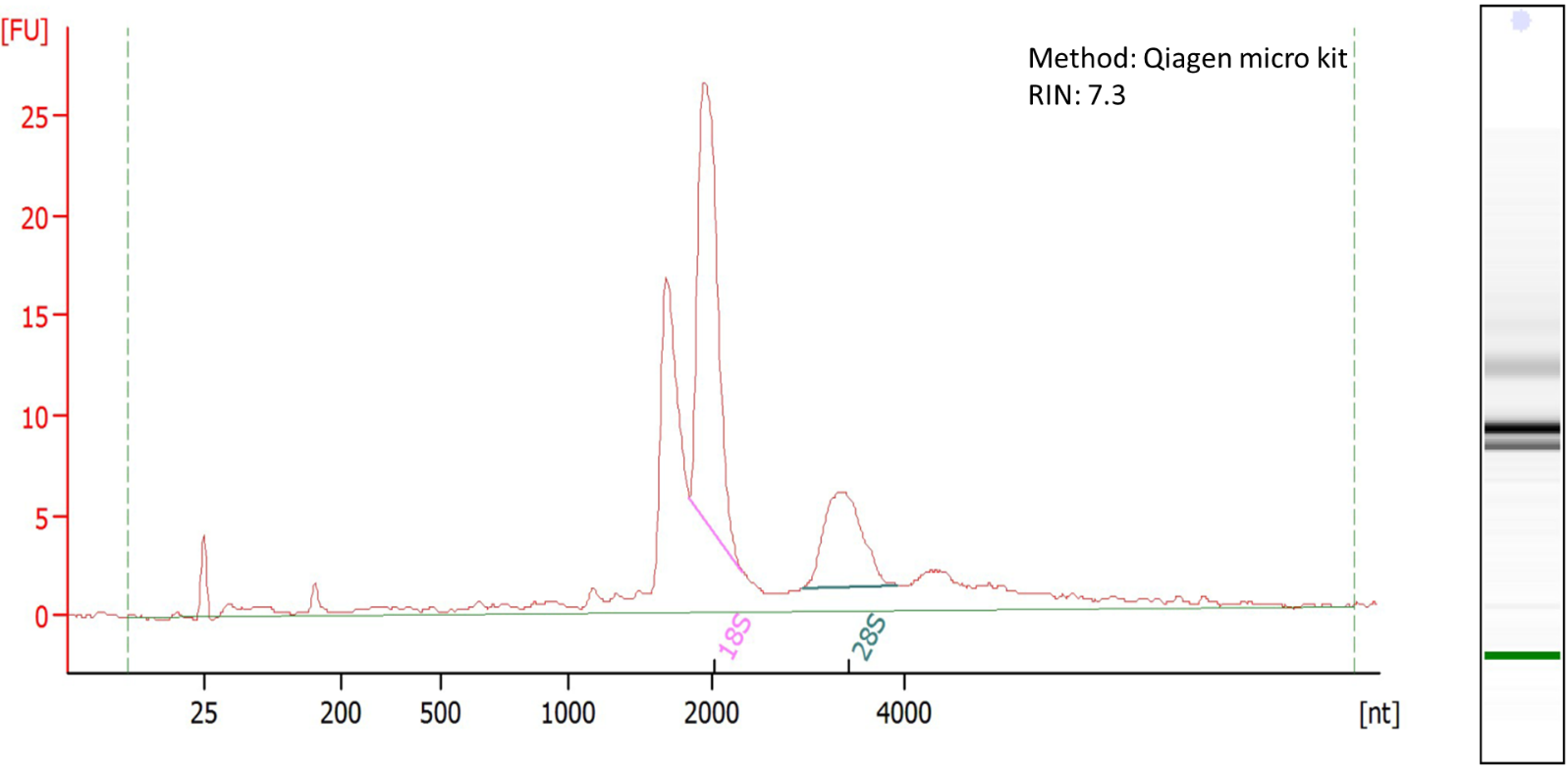


**Figure S6.** Electropherograms generated from *Moina micrura* RNA isolated using Qiagen Micro Kit method. The integrity of RNA (RIN value) was measured by Agilent 2100 Bioanalyzer Agilent Technologies. The bioanalyzer instrument used Agilent RNA 6000 Pico Kit and supplied by Agilent Technologies. X –axis units in nt (Nucleotides); Y –axis units in FU (Fluorescence Units).


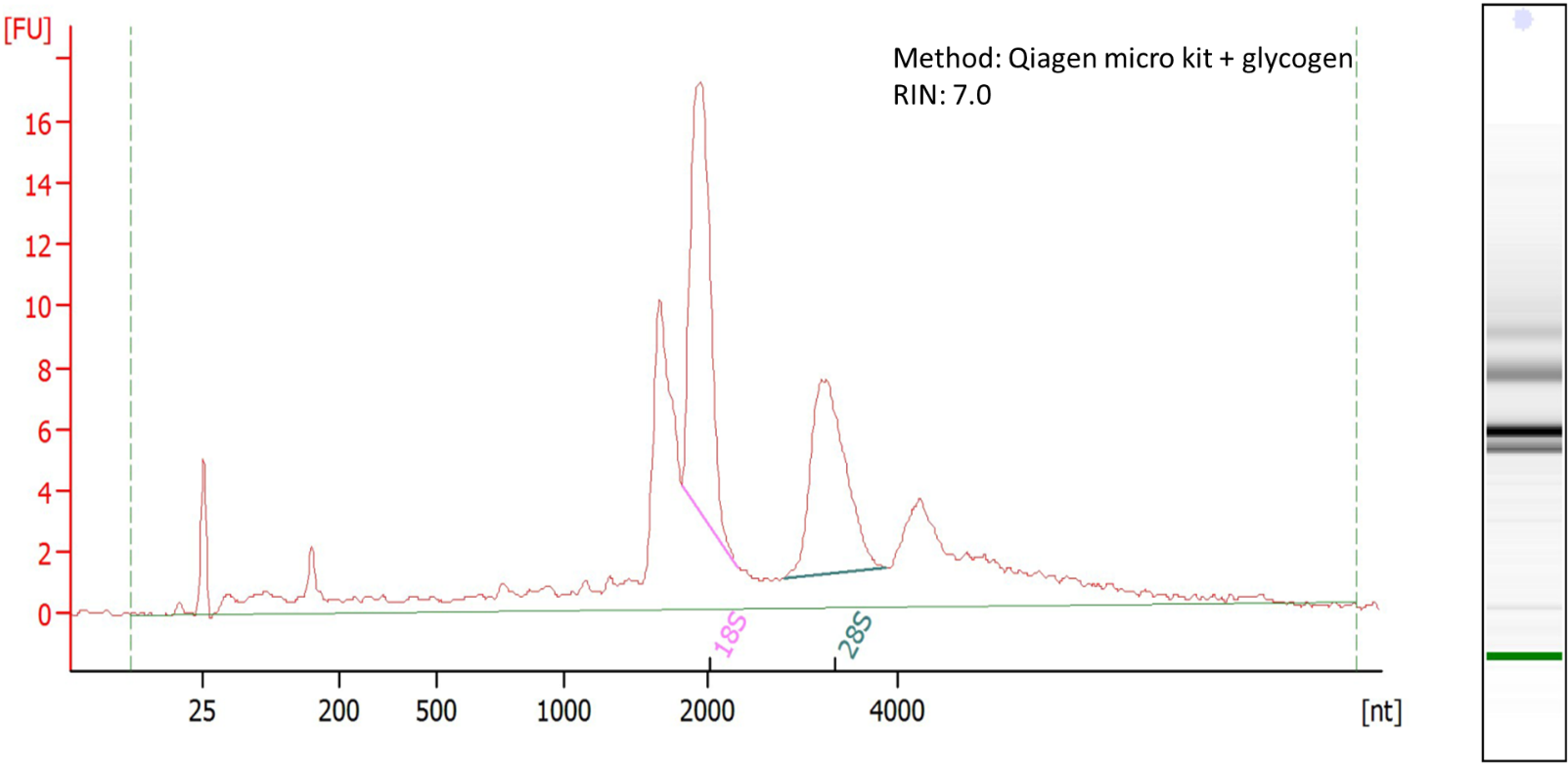


**Figure S7.** Electropherograms generated from *Moina micrura* RNA isolated using Qiagen Micro Kit + glycogen method. The integrity of RNA (RIN value) was measured by Agilent 2100 Bioanalyzer Agilent Technologies. The bioanalyzer instrument used Agilent RNA 6000 Pico Kit and supplied by Agilent Technologies. X –axis units in nt (Nucleotides); Y –axis units in FU (Fluorescence Units).
